# Feasibility of Synchronous Liver Metastasectomy During Emergency Colorectal Surgery: A Case Report

**DOI:** 10.7759/cureus.59625

**Published:** 2024-05-04

**Authors:** Angelos Cherouveim, Nektaria Dimitra Doutsini, Georgios Tzikos, Aikaterini Smprini, Konstantina Katsiafliaka, Alexandra-Eleftheria Menni, Angeliki Vouchara, George Chatziantonniou, Aristeidis Ioannidis

**Affiliations:** 1 Department of Surgery, AHEPA University Hospital of Thessaloniki, Thessaloniki, GRC

**Keywords:** synchronous tumor, oncology, hepatic metastasis, emergency, colorectal cancer

## Abstract

Colorectal cancer (CRCa) is the most frequent gastrointestinal (GI) malignancy, while the liver is the most common site of distant metastases from CRCa, arising from hematogenous spread mainly via the portal venous system. The multiform nature of tumor presentation necessitates a comprehensive approach to diagnosis, perioperative care, and oncological treatment strategy. Herein, we present a case of a 76-year-old male patient diagnosed with obstructive bowel ileus due to a sigmoid tumor with synchronous, suspicious for metastasis, liver lesion who underwent Hartmann’s sigmoidectomy in conjunction with left lateral hepatic resection at the same time. Intraoperatively significant blood loss occurred, while the postoperative course of the patient included pulmonary embolism (PE) six days after the procedure, being discharged on postoperative day (POD) 21. After oncological consensus, the patient underwent adjuvant chemotherapy and his reevaluation nine months after surgery confirmed that he is free of active disease. It is evident, however, that the number of existing studies concerning synchronous metastasectomy alongside CRCa resection in an emergency setting is limited and the literature gaps on this matter emphasize the need for further research.

## Introduction

Colorectal cancer (CRCa) is the second most common malignancy and most common gastrointestinal (GI) tumor. A total of 5.55% of the population will suffer from CRCa, with a peak incidence between 55 and 75 years of age [1]. Colonic cancer can spread through multiple different directions, including local, lymphatic, or hematogenous routes, but also transoelomically around the peritoneum. The liver is the most common site of distant metastasis from CRCa, arising mainly from hematogenous spread via the portal venous system. The likelihood of hepatic metastasis is associated with lymph node involvement, tumor size, and grade [2]. Of the patients with systemic disease, approximately 15% will have metastases at the time of diagnosis limited to the liver and only 20% of those will be eligible for resection.

Metastatic CRCa is associated with extremely limited survival. However, following successful resection of the metastatic lesion, survival is greatly improved when compared to patients who do not undergo surgery (five-year survival: 20-40% vs 15%, respectively) [3]. It is worth mentioning that in case an unexpected metastatic disease is encountered at the time of a laparotomy, the decision about whether to proceed with resection of the primary tumor depends on the volume of the distant disease, the location and the size of the primary tumor, the operation required for the effective primary tumor removal, and the operative approach. If the metastatic disease is of low volume (isolated and/or potentially resectable liver lesions) and the resection of the primary tumor is straightforward (segmental abdominal colectomy), an option could be that of resection [3]. On the other hand, in the case of systematic disease (carcinomatosis), especially if the primary tumor is minimally symptomatic, the operation should be aborted in order to facilitate early systemic neoadjuvant chemotherapy [3].

Regarding the emergency manifestation of CRCa, 20% of cases will present to the Emergency Department (ED) due to CRCa complications including intestinal obstruction, hemorrhage, or peritonitis [2]. In these instances, when compared to elective resection, the emergency or even urgent nature of the condition is associated with a worse prognosis (postoperative mortality 4.6% vs 16%), as well as impaired overall long-term survival [4,5]. This is largely due to the advanced and aggressive cancerous growth formation, strongly related to the late detection [6]. In this study we present a case of simultaneous resection of an isolated secondary metastatic hepatic lesion in the setting of an emergency Hartmann’s sigmoidectomy due to obstruction, aiming to raise awareness on the limited amount of data regarding the survival benefit of an emergency metastasectomy and to shed light on the safety and efficacy of this procedure.

## Case presentation

A 76-year-old male presented on the 15th of July 2023 to the ED of AHEPA University Hospital of Thessaloniki, Macedonia, Greece with intermittent abdominal pain and constipation spanning over the period of a week. The patient’s medical record included an ED visit to another tertiary hospital two days prior, complaining of similar symptomatology, where the laboratory results showed an acute increase in C-reactive protein (CRP) (Table 1) and the computed tomography (CT) scan performed revealed both an incomplete sigmoid obstruction due to possible neoplasia as well as one metastatic hepatic lesion. However, the patient was discharged from the aforementioned hospital with an antibiotic regimen alongside a recommendation for MRI and further gastroenterological evaluation. During our assessment, the patient’s clinical examination revealed reduced to absent bowel sounds on auscultation and mild diffuse abdominal tenderness on palpation, while on inspection cachexia was profound due to reported unintentional weight loss of about 15 kg during the last 40 days, without a conscious amendment in diet or exercise routine.

**Table 1:** Patient’s laboratory results. POD, postoperative day, the day is reported within the parenthesis; CRP, C-reactive protein; WBC, white blood cells; PCT, procalcitonin; Hb, hemoglobin; N/A, not applicable

Parameter	2 days before admission	POD (0)	POD (3)	POD (6)	POD (16)	Reference range
CRP (mg/dl)	80	7.77	24.80	N/A	N/A	<0.5
WBC count (K/μL)	N/A	13.79	N/A	N/A	N/A	3.8-10.5
Neutrophils (%)	N/A	82.5	N/A	N/A	N/A	45-74
D-dimer (ng/mL)	N/A	1170	N/A	3224	N/A	<500
PCT	N/A	N/A	2.51	N/A	N/A	>2 very likely systemic bacterial infection
Troponin T-HS (pg/mL)	N/A	N/A	N/A	43	N/A	<14
Hb (g/dL)	N/A	N/A	N/A	N/A	7.9	14-18

**Figure 1:**
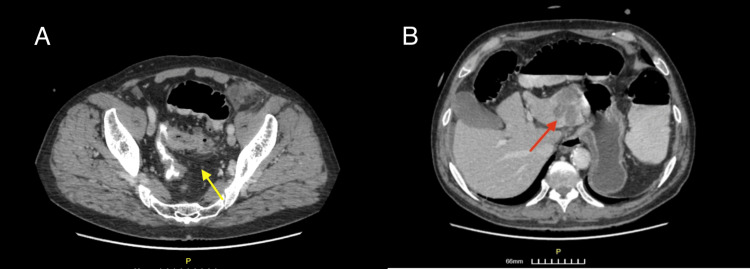
Contrast-enhanced CT after per os and IV contrast administration. Axial images. A. Bowel obstruction due to colonic neoplasia (yellow arrow), with segmental thickening and presence of diverticula. B. Hepatic metastatic lesion (red arrow). Air-fluid levels are also evident.

The patient’s laboratory results showed an increased CRP, mild leukocytosis (increased white blood cell count) with neutrophilia, and an acute elevation of D-Dimer (Table 1). The thoraco-abdominal X-ray revealed air-fluid levels necessitating the need for an abdominal CT scan. Thus, the abdominal CT scan revealed segmental thickening of the sigmoid colon (5.3 cm in length), indicative of neoplasia, causing a complete mechanical obstruction (Figure 1A). Moreover, a small collection of fluid around the thickening, pathologic regional lymph nodes, and diverticula was seen (Figure 1). In addition, on the left hepatic lobe, a hypodense lesion (44x42x36 mm) was evident, compatible with secondary metastasis (Figure 1B).

**Figure 2:**
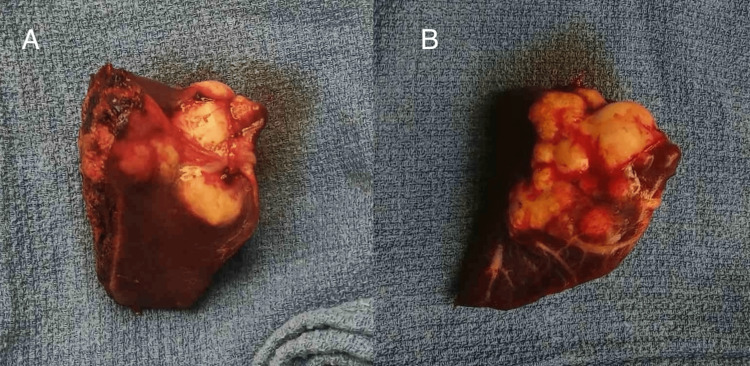
Specimen of the resected hepatic lesion from the left lateral hepatic lobe. A. Posterior view. B. Anterior view.

**Figure 3:**
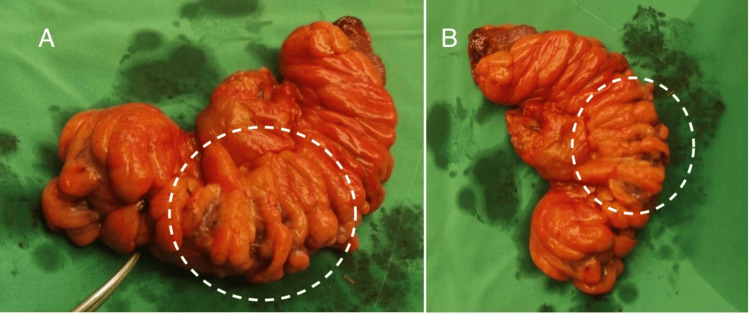
Specimen of the resected sigmoid colon. A. Anterior view. B. Posterior view. The area of malignant growth is indicated within the dotted circle.

After an informed consent was signed, the patient was driven urgently to the operating room for exploratory laparotomy during which the sigmoid tumor along with the metastatic hepatic lesion were confirmed. Thus, Hartmann’s sigmoidectomy with left lateral hepatic lobectomy (Segment II) was performed (Figures 2, 3). It is worth noting that during the hepatectomy massive bleeding occurred due to left hepatic vein rupture, resulting in about one-and-a-half-liter blood loss. The bleeding was controlled by means of hepatic sutures and hemostatic agent (SURGICEL™ Absorbable Hemostat) application while two units of packed red blood cells (pRBC) were transfused.

Postoperatively the patient was transferred intubated to the ICU for close-monitoring and mechanical ventilation alongside vasopressor supportive therapy. Based on the laboratory findings on postoperative day (POD) 3 confirming a dramatic elevation of CRP and a procalcitonin highly indicative of systemic bacterial infection (Table 1), aggressive antibiotic treatment was commenced. On the 5th POD, the patient exhibited hemodynamic stability and ventilatory improvement and thus he was extubated the same day.

However, the next day (POD 6) manifestations of desaturation, tachypnea, and dyspnoea were noted. It is worth mentioning that low molecular weight heparin administration was withheld due to intraoperative bleeding for two days postoperatively and was initiated on POD 3 (enoxaparin 1 mg/kg twice daily). Further laboratory tests recorded an acute elevation of troponin T-HS and a dramatic increase of D-Dimer (Table 1). The clinical setting was suspicious for pulmonary embolism (PE) and, therefore, a CT pulmonary angiogram (CTPA) was performed immediately. The CTPA showed a filling deficit in the right main branch of the pulmonary artery as well as in the first-class branches and the left lung lingula confirming PE. As a result, the patient was transferred to the coronary care unit (CCU) for proper treatment with anticoagulants and close monitoring. After a four-day stay in the CCU, the patient was transferred back to the surgical unit with a treatment scheme including continuation of low-molecular-weight heparin in therapeutic dosages and respiratory physiotherapy with end-goal to decrease the oxygen therapy. There, at the POD 16, a noticeable decrease in hemoglobin was noted (Table 1) and one unit of pRBC was given.

After that, the postoperative course was uncomplicated until the patient displayed symptoms of organic psychosis, including visual and auditory hallucinations, violent behavior, and self-harm ideation for which benzodiazepines, haloperidol, and other antipsychotic medication were initiated after the psychiatric evaluation was performed. The patient recovered fully and unimpededly and the remaining postoperative course was uneventful. So, 29 days after the emergency operation, the patient was discharged safely from the hospital. The aforementioned long hospital stay may be the consequence of the complicated surgical intervention causing extensive loss of blood, longer intraoperative time, and greater surgical stress. However, no safe conclusions regarding the causative nature of our intervention can be drawn.

The histology report revealed the presence of infiltrating colorectal adenocarcinoma, not otherwise specified, moderately differentiated, with lymphatic and venous invasion (V1). More specifically, three out of the total 20 lymph nodes dissected were metastatic, while in two of them, extra-lymphatic diffusion was also identified. Furthermore, the resected hepatic lobe (Section II of the liver) showed a wide area of metastasis from the colorectal adenocarcinoma. The above histological findings, based on the pTNM staging system (8th Edition AJCC), are classified as pT4apN1bpM1. Another noteworthy fact is that the histology report revealed that at the area of a diverticulum, there was colonic rupture due to necrosis of the intestinal wall accompanied by severe focal infection and abscess formation, which could justify the sepsis initiated relatively early (POD 3) during the postoperative course, that ultimately resulted in septic shock and multiorgan dysfunction.

After oncological consensus, further investigation was recommended, which included molecular profiling of the solid tumor (Tables 2, 3, 4) as well as immunohistochemical screening for the detection of protein expression of MMR genes in order to achieve the most appropriate treatment scheme. More precisely, strong nuclear positivity was observed in the majority of neoplastic cells for MSH2, MSH6, MLH1, and PMS2 proteins (as a positive internal marker lymphocyte, stromal cells, and normal mucosa of the colonic mucosa).

**Table 2:** Genetic changes detected with the Oncomine™ Precision Assay GX (total 3159 changes). SNVs, single nucleotide variants; CNVs, copy-number variants

SNVs and indels in 46 genes					CNVs, 14 genes		Fusions and expression imbalance, 16 genes		Intragenic rearrangements, 3 genes
AKT1	CHEK2	FGFR3	KIT	NTRK3	ALK	FGFR1	ALK	NTRK1	AR
AKT2	CTNNB1	FGFR3	KRAS	PDGFRA	AR	FGFR2	BRAF	NTRK2	EGFR
AKT3	EGFR	FLT3	MAP2K1	PIK3CA	CD274	FGFR3	ESR1	NTRK3	MET
ALK	ERBB2	GNA11	MAP2K2	PTEN	CDKN2A	KRAS	FGFR1	NUTM1	-
AR	ERBB3	GNAQ	MET	RAF1	EGFR	MET	FGFR2	RET	-
ARAF	ERBB4	GNAS	MTOR	RET	ERBB2	PIK3CA	FGFR3	ROS1	-
BRAF	ESR1	HRAS	NRAS	ROS1	ERBB3	PTEN	MET	RSPO2	-
CDK4	FGFR1	IDH1	NTRK1	SMO	-	-	NRG1	RSPO3	-
CDKN2A	FGFR2	IDH2	NTRK2	TP53	-	-	-	-	-

**Table 3:** Technical characteristics of the sample analyzed (Genexus system data).

Target coverage		Amplicon summary			
Base coverage at 100X	Base coverage at 350X	Average base coverage depth	Uniformity of base coverage	Percent reads on targets	Uniformity of amplicon coverage
100.00%	100.00%	3169	98.95%	93.57%	99.20%

**Table 4:** Characteristics of genetic changes detected in the sample. *highest confidence is 0 VAF, variant allele frequency; COSMIC, the catalogue of somatic mutations in cancer

Gene	Locus (GRCh 37)	Amino acid change	Nucleotide change	Allele frequency (VAF%)	Coverage	Variant ID (COSMIC database)	P-value (quality metric)*
PIK3CA	chr3:178952085	p.H1047R	c.3140A>G	24.1	2086	COSM775	0
BRAF	Chr7:140453155	p.D594N	c.1780G>A	19.5	1546	COSM27639	1.2823E-166

## Discussion

Here, we have presented the case of a 76-year-old male patient diagnosed with obstructive sigmoid cancer with synchronous liver metastasis who underwent Hartmann’s sigmoidectomy in conjunction with a simultaneous left lateral hepatic resection on an urgent basis.

Surgical excision of malignant hepatic growth, metastatic in nature with colorectal origin, should only be performed with curative effect in mind and ensure the absence of, or at the very least, minimal macroscopic residual cancerous fragments. Pre-operative assessment is essential to determine which patient is suitable for hepatic resection, based on their ability to tolerate the extent of blood loss associated with such surgical procedure, exclude the possibility of unresectable liver disease, and depict/outline the anatomy of metastases [7]. Evaluating patient appropriateness is a joint effort between the cancer multidisciplinary team and the surgeon who work together to verify that not only sufficient liver parenchyma is maintained to prevent postoperative liver insufficiency but also positive margin excision is performed, with a view to minimizing the chances of disease recurrence [8]. The metastatic hepatic lesion of our patient was resected with a 1.5 cm free margin, ensuring adequate tumor clearance and prevention of malignancy reappearance.

If unexpected metastatic disease is encountered at the time of a laparotomy, however, the situation is different. More so if it involves emergency surgery. Wong et al. reported that the primary condition necessitating emergency surgery is bowel lumen obstruction (78%) as was the case with our patient, whose colorectal carcinoma was complicated by an obstructed sigmoid lumen that ultimately caused mechanical bowel ileus [4]. The decision in this case to proceed with synchronous metastasectomy is based upon the extent, location, and size of the primary tumor as well as the operative approach [3]. As mentioned above, if the metastatic disease is low volume and the resection of the primary tumor is straightforward, it is reasonable to proceed with resection. Additionally, it is important not to biopsy potentially resectable hepatic metastases as this may cause tumor dissemination [2].

Many studies confirm that emergency colorectal resection carries a greater risk and lengthier hospital stay when compared to an elective operation. Biondo et al. reported a significantly higher early postoperative mortality rate after emergency colorectal resection (15.3%) when compared to elective (4.8%) [9]. Indeed, that was valid for our patient as well whose extended intraoperative blood loss postoperative PE in conjunction with organic psychotic syndrome led to an increase in hospitalization time to approximately one month following the emergency operation. Despite the undeniable evidence, there are certain circumstances that can modify the final outcome. For instance, an expert surgeon when combined with a medically fit patient reduces the overall mortality risk to levels closer to that of elective Hartmann’s sigmoidectomy of an uncomplicated cancer at the same stage [10].

Our patient has successfully undergone adjuvant chemotherapy and his reevaluation nine months after surgery has confirmed that he is free of active disease. Wanebo et al. have concluded that patients with an untreated solitary liver metastasis had a median survival of 19 months but none reached the five-year survival point, while patients with a resected single liver metastasis had a median survival of 36 months with five-year survivors amounting to 25%. In the majority of cases, solitary hepatic metastatic resection can be accomplished by simple excision or wedge resection, although a potential lobectomy should not be excluded [11]. Unfortunately, there are only a few retrospective studies that have compared the survival of patients with untreated potentially resectable metastases to patients who had secondary hepatic malignancy resection. However, those that do exist fortify the importance metastasectomy has on the survival of the patient [11,12].

This evidence makes hepatic metastasectomy a viable candidate even in emergency situations provided that the surgery is placed in the hands of experienced and capable surgeons [13]. Since no superior curative alternative is available at the moment (liver transplant has no evidence to prove its efficacy in metastatic liver diseases), an aggressive wide-margin surgical approach may on occasion be justified to achieve definite tumor control and prevention of disease relapse [14,15]. Even though the extent of liver resection is not a prognostic factor on its own, the goal of surgery for liver metastases is to remove all the metastatic sites where possible with a free clearance margin equal to more than 1 cm. Performing a proper wide-margin excision is of paramount importance since leaving remnants of intrahepatic metastases after a synchronous metastasectomy will not have a positive effect on overall patient survival. Accurate localization and precise determination of secondary tumor location by means of a CT scan or transabdominal ultrasound is usually performed prior. The pre-operative thoraco-abdominal CT we performed on our patient confirmed bowel obstruction due to colonic neoplasia, segmental thickening, and the presence of diverticula, and proved helpful in the localization of an isolated hepatic metastatic lesion (Figure 1). Imaging will in most cases correctly identify colorectal metastases and assess patients’ suitability for liver resection. As someone could easily deduce, the interpretation of these results should take place faster in cases of complicated CRCa constituting an emergency [15,16]. During laparotomy, a careful exploration of the abdominal cavity and intra-operative assessment is performed. The presence of metastatic lymph node infiltration in the porta hepatis and the coeliac region considerably worsens the prognosis but should not be an absolute contraindication to resection [16]. Extrahepatic disease is the only absolute contraindication to resection. Liver resection should, therefore, be considered standard therapy for all fit patients with colorectal metastases isolated to the liver [17].

Special attention should also be paid to ensure that the hepatic functional reserve is sufficient to allow adequate postoperative liver function. If remnant hepatic parenchyma is normal, since the liver has regenerative properties, up to 75% of the volume (six to eight anatomical segments) can be resected without instigating postoperative liver insufficiency [7]. In our operation, only one anatomical segment was excised ensuring that postoperative liver dysfunction is averted. CT scan volumetry has the capacity to evaluate the volume of the cancer-free parenchyma following hepatic resection. Postoperative liver function is shown to not be altered in case of residual liver volume/body >0.5% [7].

The prognosis of patients after colorectal carcinoma excision with synchronous metastasectomy localized in the liver proves to be a challenging task. Patients with only one or two liver lesions, a 1 cm excision margin, and low carcinoembryonic antigen (CEA) levels have a five-year disease-free survival rate of more than 30% [18]. Nordlinger et al. proposed a simple prognostic scoring system for use in patients after liver metastasectomy. Giving one point to each of the following factors: age, CEA level, stage of the primary tumor, disease-free interval, number of liver nodules, and resection margin >1 cm or < 1 cm, three risk groups were created with distinct two-year survival rates: 0-2 (79%), 3-4 (60%), and 5-7 (43%) [16]. The sex and the site of the primary tumor do not seem to influence patient survival. Neither does the type of resection or blood transfusions since they could reflect the surgical difficulties faced with large and numerous lesions or the added difficulty of an emergency operation. A free margin of at least 1 cm slightly alters patient survival with 30% five-year survival when the margin was greater than 1 cm, 15% when it was less than 1 cm, but 0% when resection was incomplete [18,19].

Concerning postoperative complications, most recent studies depict that in-hospital mortality ranges from 0% to 5% and is heavily influenced by perioperative blood loss, pre-operative liver function, and extent of liver resection. Postoperative complications are observed in about 25% of patients. Morbidity after hepatic resection is usually related to transient liver failure, hemorrhage or improper blood transfusion therapy, subphrenic abscesses, or biliary fistula [15-17,20]. In our case, the patient lost approximately one and a half liters of blood, which was significant and required urgent blood product transfusion. In addition, his malignancy-associated condition alongside the operation in itself, which are both severe risk factors for venous thromboembolism, led to thrombus formation ultimately causing PE [21]. However, synchronous colorectal and hepatic resection is still considered safe and efficient in treating patients with CRCa and simultaneous hepatic metastasis. By avoiding a second laparotomy, there is an overall complication rate reduction without a change in intraoperative mortality [22-24].

## Conclusions

Our case report shed light on the difficult nature but also significance of colorectal resection with synchronous hepatic metastasectomy in an emergency setting, adding another piece of data to the limited existing information on this field. The multifaceted nature of tumor presentation requires a comprehensive approach to diagnosis, necessitating extensive surgical knowledge and experience. Future studies can delve deeper into optimal emergency resection protocols, aid in the establishment of a simple prognostic scoring system, and help refine postoperative care. By addressing these gaps, we can strengthen our ability to provide safe and effective treatment to patients with secondary liver metastases, requiring urgent emergency resection.
